# Expression of Osteoprotegerin in Placenta and Its Association with Preeclampsia

**DOI:** 10.1371/journal.pone.0044340

**Published:** 2012-08-30

**Authors:** Pei Shen, Yunhui Gong, Tao Wang, Yueyue Chen, Jin Jia, Shanshan Ni, Bin Zhou, Yapin Song, Lin Zhang, Rong Zhou

**Affiliations:** 1 Department of Obstetrics and Gynecology, West China Second University Hospital, Sichuan University, Chengdu, China; 2 Laboratory of Molecular and Translational Medicine, West China Second University Hospital, Sichuan University, Chengdu, China; Institute of Zoology, Chinese Academy of Sciences, China

## Abstract

**Background:**

Osteoprotegerin (OPG), a key regulatory factor in bone metabolism, was documented also a potential pro-angiogenic factor, which acts an important role in protecting vascular endothelial cells. Since preeclampsia has gradually been employed to be vascular diseases, we speculated that OPG might be associated with preeclampsia. The study was to evaluate the level of OPG protein and mRNA in placenta, and investigate the relationship between OPG and the pathogenesis of preeclampsia.

**Methodology/Principal Findings:**

Placental specimens from 30 term normal pregnancy, 30 severe preeclampsia and 30 mild cases were studied. The expression and levels of OPGs’ protein and mRNA were detected by immunohistochemisty, western blot analysis and real-time quantitative PCR analysis respectively. The expression of OPG protein was found in cytoplasm of placenta cytotrophoblasts and syncytiotrophoblasts in three groups. There were no significant differences of OPG protein between the maternal and fetal side in each group. The OPG protein and mRNA levels in severe preeclampsia were significantly higher than those in mild cases and normal pregnancy. However, there were no markedly differences of the OPG protein and mRNA levels between term delivery and preterm delivery in severe cases. In preeclampsia, the OPG protein and mRNA level was positively correlated with systolic blood pressure and 24 h urinary protein respectively.

**Conclusions/Significance:**

OPG protein and mRNA level in placentas of preeclampsia were found abnormal compared with normal pregnancy. In preeclampsia, the OPG protein and mRNA levels were closely related with its important clinical parameters. Taken together, OPG might be closely correlated with the pathogenesis of preeclampsia.

## Introduction

Preeclampsia is a specific disorder known to promote maternal or perinatal mortality and morbidity during pregnancy [1]. A large of evidences suggested that preeclampsia could be associated with many factors, such as endothelial dysfunction, inflammation, insulin resistance [2]–[4], although its etiology and pathogenesis has not been extensively investigated. Interestingly, nowadays researches indicated the endothelial dysfunction may potentially function as a inducer role in the pathogenesis of preeclampsia [5], [6], [7]. Osteoprotegerin (OPG), one of the superfamily members of the tumor necrosis factor receptors, which can regulate both bone absorption and inhibit osteoclast maturation, is a key regulatory factor in bone metabolism [8], [9]. Recently, many studies documented that OPG was also a potential pro-angiogenic factor, which acts as an important regulatory factor in protecting vascular endothelial cells [10], [11], [12]. Price demonstrated that OPG had ability to reduce the calcification of arteries in animal models [11]. Kobayashi-Sakamoto indicated that OPG contributed to the survival of human microvascular endothelial cells during periodontitis [12]. In addition, Pritzker showed that OPG had roles in endothelial cell survival and the prevention of arterial calcification in human [12]. Therefore, OPG has been widely studied in the vascular-related diseases, such as coronary heart disease [13], [14], diabetes [15], [16], [17], high blood pressure [18] and peripheral artery diseases [19]. Since preeclampsia has gradually been employed to be vascular diseases during pregnancy, and endothelial dysfunction maybe involved in its pathogenesis, we speculated that OPG might be also associated with preeclampsia.

Hence, in attempting to validate the effects of OPG on vascular to provide a solid foundation for future preeclampsia studies, here, we evaluated the expression of OPG in placenta for its putative properties.

## Materials and Methods

### Participants and Placenta Collection

All the samples were obtained from the Department of Obstetrics & Gynecology, West China Second University Hospital, Sichuan University, during the period from November 2008 to July 2009. Preeclampsia was defined as blood pressure >140/90 mmHg on 2 separate occasions 6 hours apart or a single recording of a diastolic pressure of ≧110 mmHg, in association with proteinuria ≧1+ on dipstick testing or proteinuria ≧300 mg per 24 hours after 20 weeks’ gestation [20]. Totally sixty women with preeclampsia were recruited and divided into two groups, in which include 30 severe cases of preeclampsia (9 term delivery and 21 preterm deliveries, 15 primipara and 15 multipara), 30 mild cases of preeclampsia (all term delivery, 24 primipara and 6 multipara) and 30 normal pregnancies as negative control (normotensive term pregnancies, 23 primipara and 7 multipara). In all the participants, hemolysis, elevated liver enzymes, low platelet count (HELLP syndrome) was excluded [20].

The study was approved by the Institutional Ethics Committee of West China Second University Hospital, and all patients were provided with written informed consent. All the patients delivered undergoing elective cesarean section. The indications for cesarean section included previous cesarean section, breech presentation and social indications. Exclusion criteria included multiple pregnancy, diabetes, chronic nephritis, chronic hypertension, heart diseases and fetal malformation.

Information on demographic characteristics of all the participants was recorded. Gestational age was based on the last menstrual period and/or was confirmed by ultrasound examination conducted in the first trimester.

**Table 1:** Demographic characteristics of the subjects.

group	N	Maternal age (year)	Gestational weeks of delivery	Birth weight(g)	Length of babies(cm)	Prepregnancy BMI(kg/m^2^)
normal	30	31.93±4.60	38.87±0.71	3262.70±324.01	48.90±2.09	21.71±3.25
mild cases	30	30.53±4.78	38.54±1.01	3166.60±311.93	48.52±1.55	22.26±3.25
Severe cases	30	31.57±5.18	35.31±3.05* 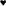	2412.00±766.99* 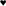	45.29±3.48 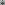 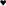	22.18±2.00

Abbreviation: BMI, body mass index.

Values are given as mean ± SD unless otherwise indicated.

* *p* = 0.000,

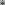
p = 0.002 for severe cases vs mild cases or normal group.

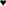
 *p* = 0.000 for severe cases vs normal group.

### Specimen Collection

Placental tissues were collected as described previously [21]. Placental tissues were collected immediately after delivery. Tissue biopsies of approximately 1.0 cm3 (Avoiding vessels and/or calcium deposits) in the center of the placenta were taken from both the fetal and the maternal side. The specimens, including controls, used in our study were preserved and stored by the Tissue Bank Core Facility at Sichuan University. Samples were washed with normal saline three times, fixed in 10% formaldehyde solution, then routinely paraffin-embedded and sliced. Additional placental samples were collected and stored at −80°C for subsequent analyses.

**Figure 1:**
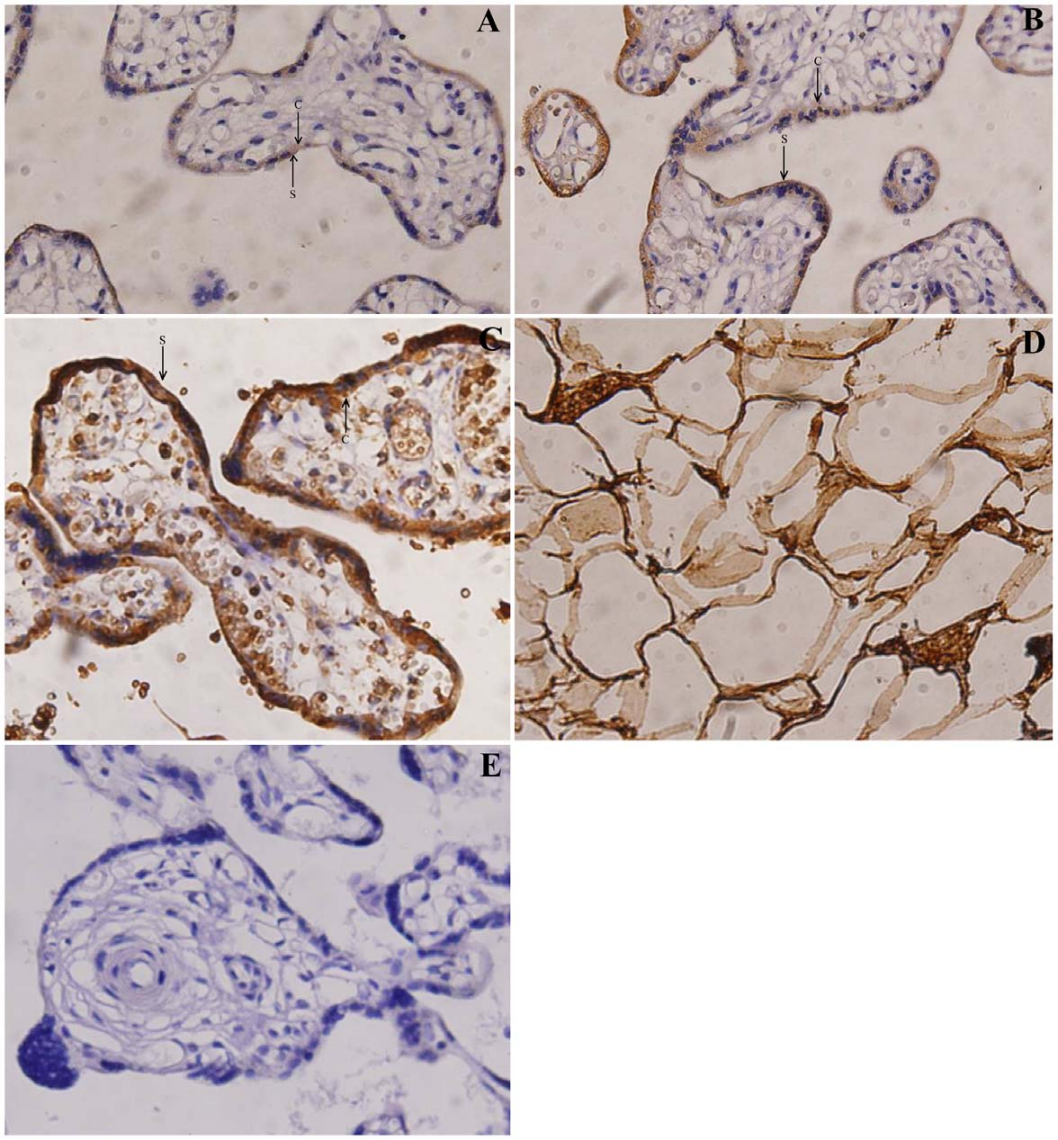
Immunoreactivity for OPG on human placental tissues. Immunoreactivity for OPG on normal term placentas (A), mild preeclampsia placentas (B), and severe preeclampsia placentas (C). OPG was localized in cytotrophoblast [C] and syncytiotrophoblast [S] cells (×400). Positive control of adipose tissues shows OPG immunostaining (D; ×400); however, negative control of the primary antibody normal term placenta obtained by substituting the primary antibody with phosphate buffer saline (PBS) shows no OPG immunostaining (E; ×400).

### Immunohistochemistry Analysis

Placental tissue samples from the maternal side and fetal side were first incubated with the mouse anti-human OPG monoclonal antibody (Novus Biologicals, USA, No. NBP1-39984, 1∶1000) overnight at 4°C prior to a 20 minutes 5% hydrogen peroxide treatment. Samples were subsequently incubated with Envision/HRP-conjugated goat anti-mouse for 30 min at 37°C followed by a brief 5 minutes of 3,3′-diaminobenzidine incubation. Treated sections were routinely washed by 0.1 M phosphate buffer (pH 7.4) with 5 minutes interval. Finally, sections were washed in distilled water. Counterstaining was performed using hemotoxylin.

Adipose tissue from the subcutaneous tissues of the rat was used as a positive control. Negative control was performed by replacing the mouse anti-human OPG monoclonal antibody with phosphate buffer saline (PBS) in the same condition. The Brown-yellowish cytoplasm indicated the positive result under the optical microscope.

**Figure 2:**
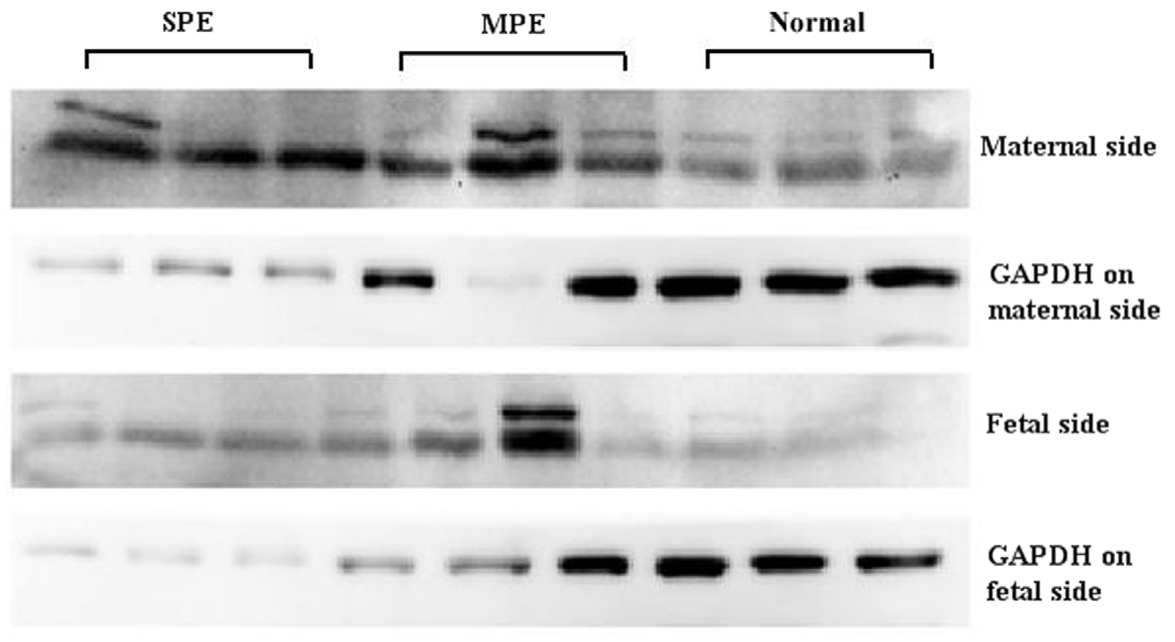
Western Blotting analysis result of OPG protein expressions in different groups.

**Figure 3:**
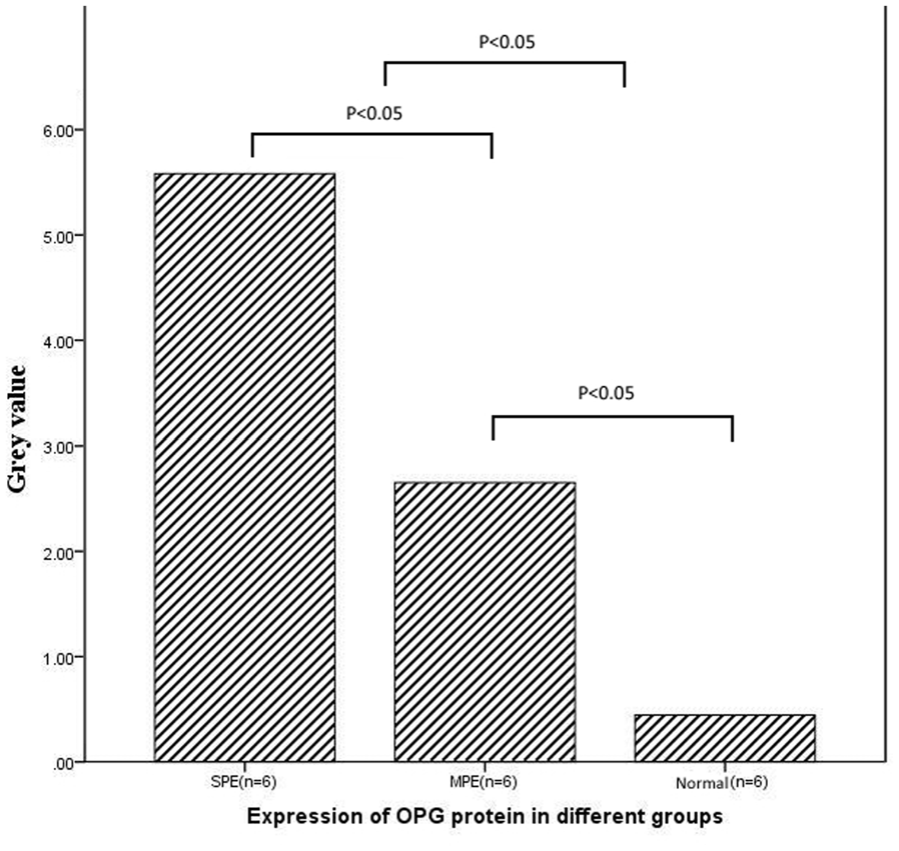
Expressions of OPG protein in different groups (Gray Value).

### Western Blotting

20–50 µg of total protein was separated by 10%-15% gel gradient SDS-PAGE under reducing conditions For Western blotting protein analysis. Proteins were then transferred to a polyvinylidene fluoride (PVDF) membrane. The PVDF membrane was incubated with blocking buffer (PBS containing 5% non fat milk) for 2 h at room temperature. Primary antibody was mouse anti-osteoprotegerin (Novus, United States). Membranes were incubated overnight at 4°C. The membrane was washed twice with TBST for 10 min and incubated with secondary antibodies (Santa Cruz, United States) for 2 h at room temperature. After washing, osteoprotegerin was detected using a chemiluminescence reaction. The results were analyzed with Quantity One (BIO-RAD, United States) software. Protein levels were normalized to GAPDH protein.

### RNA Extraction, Reverse Transcription and Real-time Fluorescent PCR

Fifty milligrams of the placental maternal side tissues from each sample were processed. Total RNA was extracted according to manufacture’s instructions (Tiangen Biotech, Beijing, Co, Ltd, China) and was described previously [21].

Real-time PCR analyses were performed in a fluorescent temperature cycler (SYBR Green dye (Tiangen Biotech, Beijing, Co, Ltd, China)) according to the manufacturer’s instructions. Two micrograms of total placental RNA were used for each reaction. A 20-µ l amplification mixture contained 3.0 µl RT reaction products, 25 mM MgCl_2_, 1 µl of each primer, and 1 µl of each probe (Invitrogen Corporation, Carlsbad, USA). The PCR cycling conditions included an initial denaturation at 95°C for 2 minutes, followed by 40 cycles at 95°C for 15 seconds, 64°C for 15 seconds, and 68°C for 20 seconds.

The oligonucleotide-specific primers for OPG (167 bp) and β-actin (205 bp) were fellows. For OPG, forward primer was 5′-AGGAAATGCAACACACGACA-3′, reverse primer was 5′-TACTTTGGTGCCAGGCAAAT-3′. For β-actin, forward primer was 5′-TGACGTGGACATCCGCAAAG-3′, reverse primer was 5′-CTGGAAGGTGGACAGCGAGG-3′ (Invitrogen Corporation, Carlsbad, USA). The cycle threshold value was obtained using the software provided by the manufacturer. Relative values of PCR products was normalized with respect to the β-actin level in each sample using the formula (2**^-ΔΔCt^**) [22].

### Statistical Analysis

SPSS16.0 software was used to process data. All data were expressed as the mean ± SD. Differences between groups were evaluated using one-way ANOV, followed by Mann-Whitney U test and paired or unpaired–t test and Wilcoxon signed-rank test. Non normal distribution data was analyzed with Jonckheere-Terpstra test and median test. The associations between OPG and continuous variables were analyzed by Pearson or Spearman’s correlation coefficients. Results were considered statistically significant if the ***P***-value was less than 0.05. All statistical tests were two-sided.

## Results

### The General Characteristics of Participants

Gestational week of delivery, birth weight and lengths of infants in the severe preeclamptic group were less than those of the normal group and the mild preeclamptic group (all p<0.01). There were no significant difference in maternal age and pre-pregnancy body mass index among those three groups (all P>0.05, table 1).

### The Expression of OPG Protein in Placenta

The expression of OPG was found in cytoplasms of placental cytotrophoblasts and syncytiotrophoblasts in normal pregnancy, mild and severe preeclamptic group. Adipose tissues of rat was treated as positive control and showed high expressions of OPG, whereas as negative control, there was no immunostaining showed in cytotrophoblast and syncytiotrophoblast cells ( Fig1).

Expressions of OPG protein in severe preeclamptic group (SPE), mild preeclamptic (MPE) and normal control (CTRL) group were shown in figure 2 **(** Fig2 **)** by Western blotting. No significant difference of OPG protein level was found by western blot between the maternal side and fetal side of the placenta in each group (P>0.05).On both the maternal side and the fetal side of placenta, strong expression of OPG protein was observed in severe preeclampsia group (SPE). In Mild preeclampsia group (MPE) a significant expression of OPG protein was also observed. While the expression of OPG protein remained low in normal control group. There is a significant difference between the severe preeclampsia group and the normal control group (P<0.05) **(** Fig3 **)**.

### Placental OPG-mRNA Level and its Correlation with Preeclampsia

**Figure 4:**
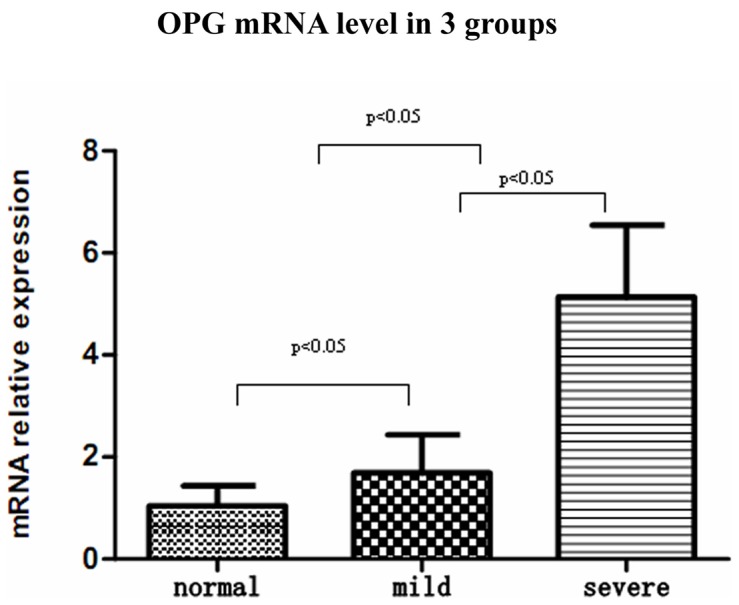
The OPG mRNA level in human placental tissues. The OPG mRNA level in severe preeclamptic group was much higher than those of the mild preeclamptic and normal control group.

OPG-mRNA of all placentas was detected in all groups. The level of OPG-mRNA in the severe preeclamptic group was significantly higher than those in the mild group and the normal group (Fig4). There was no significant difference of OPG-mRNA level between term delivery and preterm delivery from the severe preeclamptic group (5.1620±1.2220 vs 5.1307±1.5019, p = 0.967). However, OPG-mRNA level in term severe preeclamptic group was much higher than that of the normal group (5.1620±1.2220 vs 1.0535±0.3989, P<0.001).

### Correlations between Clinical Parameters and Expressions of OPG

The expression of OPG in preeclamptic group was positively correlated with either systolic blood pressure before delivery or 24-hour urine protein, while negatively correlated with plasma albumin. In addition, the level of OPG-mRNA in preeclamptic group was positively correlated with systolic, diastolic blood pressure before delivery and 24-hour urine protein, while negatively correlated with plasma albumin, gestational weeks, and neonatal birth weight or birth length. However, only OPG-mRNA level was negatively correlated with plasma albumin in normal pregnancy group (table 2,3).

**Table 2:** Correlations between clinical parameters and expressions of OPG protein or mRNA in preeclamptic group(including mild and sever preeclamptic group).

parameters	OPG protein		OPG mRNA	
	R	P	R	P
SBP pre-delivery	0.487	0.016	0.787	0.000
DBP pre-delivery	0.262	0.217	0.460	0.024
Urine protein(g/24 h)	0.529	0.008	0.742	0.000
Serum albumin(g/L)	−0.553	0.005	−0.542	0.006
Serum Cr(umol/L)	0.114	0.595	0.185	0.388
Serum BU(mmol/L)	0.359	0.085	0.199	0.35
Fast sugar()	0.250	0.239	0.327	0.119
Delivery weeks	−0.366	0.078	−0.462	0.023
Birth weight(g)	−0.269	0.081	−0.658	0.000
Birth length(cm)	−0.264	0.087	−0.605	0.000

**Table 3:** Correlations between clinical parameters and expressions of OPG protein or mRNA in normal group.

parameters	OPG protein		OPG mRNA	
	R	P	R	P
SBP pre-delivery	0.046	0.900	−0.004	0.992
DBP pre-delivery	0.310	0.383	0.021	0.953
Serum albumin(g/L)	0.269	0.453	−0.647	0.043
Serum Cr(umol/L)	0.012	0.975	0.403	0.248
Serum BU(mmol/L)	0.339	0.338	0.142	0.696
Fast sugar()	−0.034	0.927	0.554	0.097
Delivery weeks	−0.136	0.475	−0.134	0.713
Birth weight(g)	−0.171	0.367	0.400	0.252
Birth length(cm)	−0.190	0.315	0.314	0.377

## Discussion

OPG was firstly discovered by Simonet in 1997 [8], which is one of the superfamily members of the tumor necrosis factor receptors, is a key factor in bone metabolism [8], [9].Recently, many studies also documented that OPG was a potential pro-angiogenic factor, which acts as an important factor in protecting vascular endothelial cells [10], [11], [12]., and discovered that OPG was involved in coronary heart disease [13], [14], high blood pressure [18] and peripheral artery diseases [19].

It is certain that the OPG distributed in all of the organs and tissues, especially in the placentas and gestational tissues [8], [23], and placenta is an important organ to produce OPG during pregnancy [23]. Hong found that the serum OPG level in normal term pregnancy was significant higher than that of early pregnancy (6.63 pmol/L vs 3.98 pmol/L, P<0.001) [24]; during normal pregnancy, the serum OPG concentration was gradually increased from the first to third trimester, and gradually decreased from postpartum 4 days to 30 days [25]. Our research also discovered that serum OPG levels increased through normal pregnancy, with maximum level in the third trimester, and serum OPG levels in third trimester were significantly higher than those in second and first trimester(data unpublished). These studies suggested OPG were gradually changes with pregnancy.

As we know, preeclampsia is a specific vascular disease, the endothelial dysfunction maybe a crucial factor in the pathogenesis of preeclampsia and OPG is considered to protect vascular endothelial cells. Therefore, we speculated that OPG might be involved in the pathogenesis of preeclampsia. However, whether OPG is expressed in placenta with preeclampsia is still elusive and the mechanism of OPG in the pathogenesis of preeclampsia is unknown.

In our study, we demonstrated that OPG was expressed in the cytoplasm of cytotrophoblasts and syncytiotrophoblasts in both normal term and preeclamptic placental tissues. Our results were, in part, consist with Simonet and Phillips’ studies [8], [23]. In addition, the expression OPG is the same as other hormones, such as Leptin, IGF-1 and adiponectin/receptor, which are associated with the pathogenesis of preeclampsia [21], [26], [27], [28]. The roles of OPG in different cells types during pregnancy, especially in preeclampsia, are unknown and needed to be study further.

We further demonstrated OPG protein expressed in the maternal side and fetal side in placental tissues. However, no significant difference of OPG protein level between the maternal side and fetal side of the placenta in each group was observed. This result suggested that both the maternal side and fetal side of OPG might have the same special functions, although all of these cells are of fetal origin.

In addition, we assessed OPG protein and mRNA levels in placenta and its correlation with the severity of preeclampsia. Our results showed that the changes of protein and mRNA of OPG in each group were very consistency, both OPG protein and mRNA levels were significantly higher in severe cases of preeclampsia than those mild cases and normal pregnancy. Moreover, OPG protein and mRNA levels were much higher in mild preeclampsia than those in normal pregnancy. These indicated that the changes of protein and mRNA levels of OPG were more obvious in severe preeclampsia, suggesting the expression of OPG were consistent with the severity of preeclampsia.

Moreover, our study discovered that, in preeclamptic group, both the OPG protein and mRNA was positively correlated with systolic blood pressure and 24 h urinary protein, while negatively correlated with plasma albumin level; furthermore, OPG-mRNA levels was also positively correlated with diastolic blood pressure, and negatively correlated with gestational weeks, neonatal birth weight and birth length. However, in normal pregnancy, only OPG-mRNA level was negatively correlated with plasma albumin. These results suggested that OPG protein and mRNA levels in preeclampsia were closely associated with the important clinical parameters of preeclampsia, and the association was stronger between the OPG-mRNA and the clinical parameters of the cases of preeclampsia.

In summary, OPG protein and mRNA expression in placentas of preeclampsia were obviously increased compared with that of the normal pregnancy. Gestational weeks did not affect the level of OPG protein and mRNA. In preeclamptic group, the levels of OPG protein and mRNA were closely related with its important clinical parameters. The abnormal OPG might be involved in the pathogenesis of preeclampsia.
